# Electrochemical Aptamer-Based Biosensor for Detecting Pap31, a Biomarker for Carrion’s Disease

**DOI:** 10.3390/s24227295

**Published:** 2024-11-15

**Authors:** Keaton Silver, Andrew Smith, Haley V. Colling, Nico Tenorio, Teisha J. Rowland, Andrew J. Bonham

**Affiliations:** 1 Department of Chemistry & Biochemistry, Metropolitan State University of Denver, Denver, CO 80204, USA; ksilver4@msudenver.edu (K.S.); hcollin5@msudenver.edu (H.V.C.); ntenorio@msudenver.edu (N.T.); 2 Department of Biology, University of Kansas Medical Center, Kansas City, KS 66061, USA; asmith86@kumc.edu; 3 Independent Researcher, Broomfield, CO 80020, USA; teisha42@gmail.com

**Keywords:** aptamer, carrion’s disease, E-AB, electrochemical biosensor, neglected tropical disease

## Abstract

Carrion’s disease, caused by infection with the bacterium *Bartonella bacilliformis* (*B. bacilliformis*), is effectively treated with antibiotics, but reaches fatality rates of ~90% if untreated. Current diagnostic methods are limited, insufficiently sensitive, or require laboratory technology unavailable in endemic areas. Electrochemical aptamer-based (E-AB) biosensors provide a potential solution for this unmet need, as these biosensors are portable, sensitive, and can rapidly report the detection of small molecule targets. Here, we developed an E-AB biosensor to detect Pap31, a biomarker of Carrion’s disease and an outer membrane protein in *B. bacilliformis*. We identified an aptamer with Pap31-specific binding affinity using a magnetic pull-down assay with magnetic bead-bound Pap31 and an aptamer library followed by electrophoretic mobility shift assays. We incorporated the Pap31-binding aptamer into a DNA oligonucleotide that changes conformation upon binding Pap31. The resultant Pap31 E-AB biosensor produced a rapid, significant, and target-specific electrical current readout in the buffer, demonstrating an apparent K_D_ of 0.95 nM with a limit of detection of 0.1 nM, and no significant signal change when challenged with off-target proteins. This proof-of-concept Pap31 biosensor design is a first step toward the development of more rapid, sensitive, and portable diagnostic tools for detecting Carrion’s disease.

## 1. Introduction

Carrion’s disease is a neglected tropical disease endemic to Peru, Ecuador, and Colombia, caused by infection with the bacterium *Bartonella bacilliformis* (*B. bacilliformis*). Phlebotomine sandflies (*Lutzomyia verrucarum*) transmit *B. bacilliformis* through biting, and consequently, the distribution of Carrion’s disease and *L. verrucarum* are closely associated [1,2]. In some rural areas of South America, Carrion’s disease fatalities of up to 88% have been reported in untreated patients, making it a significant public health concern [1,3,4]. The high fatality rates primarily occur during the “acute phase” of the disease, also called “Oroya fever,” when erythrocyte infection can cause symptoms including fever, severe hemolytic anemia, anorexia, immunosuppression, and death [3]. Children and pregnant women are particularly affected, with children having the highest mortality rates and pregnant women being susceptible to premature births, miscarriages, and fetal deaths [3,5]. Carrion’s disease additionally includes a “chronic phase”, also called “verruga peruana”, that significantly impacts the quality of life as it is characterized by eruptive, painful, and disfiguring warty lesions (“Peruvian warts”) that may persist for up to a year [3,4]. While early diagnosis can lead to effective antibiotic treatments that greatly reduce fatality rates to ~10% [3,5], current diagnostic methods are limited.

Because Carrion’s disease symptoms are similar to those of other diseases in the affected regions, an accurate diagnosis based on clinical symptoms alone is challenging, creating a need for disease-specific diagnostic tests. Existing Carrion’s disease diagnostics include polymerase chain reaction (PCR) and quantitative real-time PCR (qPCR) tests, serological tests including Enzyme-Linked Immunosorbent Assays (ELISAs), fluorescence antibody tests, and immunoblots, all of which typically require a well-outfitted laboratory, which is usually unavailable in affected regions, particularly in impacted rural areas [3]. Even when available, techniques such as PCR require several hours to generate results [6]. The most commonly used early diagnostic method is Giemsa-stained peripheral smears, as this technique can be performed using a limited laboratory setup. However, while the specificity of this technique is high, sensitivity is so poor that diagnosis is unreliable [3]. Blood culturing has also been employed, but because this approach frequently returns false negative results and lengthy culture times are required (i.e., a mean time of 18 days), blood culturing is not a viable option [4,7,8,9]. Consequently, to improve treatment outcomes and survival for affected individuals, improved rapid, early diagnostic tools for detecting Carrion’s disease are needed.

A promising target protein, or biomarker, to use in diagnostic tools for early detection of Carrion’s disease is Pap31. Pap31 is an outer membrane protein of *B. bavilliformis*, known to be a highly expressed antigen in *B. bacilliformis* infections [10,11] and a potential seromarker for Carrion’s disease, making it appealing for blood-based diagnostics for Carrion’s disease [2,3,12]. The ability of Pap31 to be used in Carrion’s disease diagnostics has been explored in approaches including ELISAs, Western blots, and rapid lateral flow assays [10]. However, it is challenging to adapt these approaches to clinical or non-laboratory settings, such as the remote regions where Carrion’s disease is endemic.

**Figure 1:**
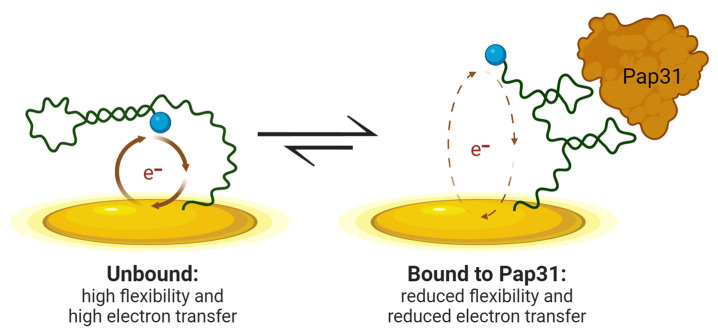
Schematic of the Pap31 E-AB biosensor. In the unbound state, the aptamer (black curvy line) possesses relatively high levels of conformational flexibility and electron transfer between the gold-plated electrode (yellow disc) and methylene blue redox group (blue ball). Upon Pap31 binding, the aptamer adopts a more rigid conformation and relatively low levels of electron transfer. Together, this results in a signal-off mechanism. Created in BioRender. Bonham, A. (2024) BioRender.com/s17v834.

Here, we present a DNA oligonucleotide electrochemical aptamer-based (E-AB) biosensor utilizing Pap31 targeting as a proof-of-concept, early-stage investigation towards the eventual development of a portable, accurate, and sensitive tool for diagnosing Carrion’s disease. E-AB biosensors have been shown to function robustly, reproducibly, and rapidly (with readouts available within minutes) to detect a target in complex fluids such as blood [13,14]. Additionally, in both our work and the work of others, E-AB biosensors display multi-hour stabilities, even with constant cycling and sensor regeneration [13,14,15]. A DNA E-AB biosensor is comprised of a target-binding DNA aptamer incorporated into a DNA oligonucleotide that changes conformation upon binding the target protein [9,16,17]. The DNA oligonucleotide additionally includes a redox reporter group (e.g., methylene blue) and is bound to a gold-plated electrode surface. When the DNA oligonucleotide undergoes a conformation change upon target binding, this alters the distance of the redox reporter group from the electrode surface, resulting in a measurable change in electrical current [18]. To develop our Pap31 E-AB biosensor design, initially, a magnetic bead-based pull-down assay was used to identify potential Pap31 binding aptamers. The identified aptamers were fluorescently labeled and analyzed in electrophoretic mobility shift assays (EMSAs) to determine the aptamer with the highest binding affinity for Pap31. To enable conformational change in the presence of Pap31, an aptamer with two isoenergetic (equally energetically favorable) conformations was generated using computational modeling. This aptamer was incorporated into an E-AB biosensor design and found to display a measurable decrease in the electrical current generated in the presence of Pap31, utilizing a “signal-off mechanism” (Figure 1). This Pap31 E-AB biosensor demonstrated high sensitivity, producing a detectable current change using concentrations of Pap31 far lower than those known to produce an immunogenic response [11] (0.1 nM vs. 100 nM, respectively). No significant change was observed in the presence of off-target proteins, which also demonstrated the specificity of the biosensor design. Together, the biosensor design presented here demonstrates the potential for the future development of a more portable, rapid, and sensitive diagnostic tool for detecting Carrion’s disease.

## 2. Materials and Methods

Unless otherwise stated, all reagents were purchased from Sigma-Aldrich (St. Louis, MO, USA).

### 2.1. Design, Transformation, Expression, and Purification of Recombinant Pap31 Protein

To produce recombinant Pap31 protein, we developed a Pap31-expressing plasmid, transformed it into *Escherichia coli* (*E. coli*), induced expression, and then purified and concentrated the protein. We designed a plasmid expressing Pap31 using the *Pap31* gene sequence from the *B. bacilliformis* strain KC583 (Genbank accession number DQ207957) and the plasmid backbone of a pET Bacterial Recombinant Protein Vector (VectorBuilder, Chicago, IL, USA; Supporting Figure S1). We transformed this plasmid into BL21-competent *E. coli*, which were inoculated into Erlenmeyer flasks containing lysogeny broth (LB) with ampicillin (10,000 mg/L) and incubated in a shaking incubator (37 °C) at 220 rpm for >12 h. We inoculated larger flask cultures using these initial cultures and continued shaking incubation under the same conditions for an additional ~5 h. We induced protein expression by adding isopropyl-β-thio-galactoside (IPTG) (0.5 mM) and continued shaking incubation for two hours. We centrifuged the resultant cell cultures (12,000 rpm for 20 min) and stored the pellet at −80 °C. We later resuspended the pellet in a denaturing lysis buffer (Urea [8M], Tris [50 mM], DTT [2 mM], and 1% Tween) and incubated the mixture with 0.1 mg/mL of lysozyme for 30 min, followed by sonication for 6 min, and DNAse incubation (10 units) for 10 min. The resultant mixture was centrifuged (21,130 rcf for 10 min), and the supernatant passed through a 0.45 µm filter and processed using fast protein liquid chromatography (FPLC). We performed FPLC using a Profinity IMAC nickel column (Bio-Rad, Hercules, CA, USA) under denaturing conditions, specifically using buffer comprised of urea (8 M), NaH_2_PO_4_ (100 mM), and Tris (10 mM), a wash buffer with the pH adjusted to a pH of 6.3, a first elution buffer with a pH of 5.9, and a second elution buffer with a pH of 4.5. Fractions with absorbance peaks of 280 nm were analyzed using 10% SDS-PAGE with a protein ladder (#P7711S; New England Biolabs, Ipswich, MA, USA) and silver nitrate staining. Using this approach, we observed an isolated band at ~31 kDa (Supporting Figure S2), the anticipated mass of the recombinant Pap31 protein. To refold the recombinant Pap31 protein into its native conformation, we dialyzed the protein using a Zeba Spin Desalting Column (ThermoFisher, Waltham, MA, USA), concentrated the sample to 250 µL in the desalting column, added 3 mL of buffer (Urea [6 M], Tris [10 mM], NaH_2_PO_4_ [100 mM]), and then reconcentrated the protein to a 250 µL total. This process of desalting and resuspension was repeated at urea concentrations of 3 M, 1 M, 0.5 M, and 0 M. We performed the SDS-PAGE as described above using the native protein, which again produced the expected single band at ~31 kDa.

### 2.2. Selection of Candidate Pap31-Binding Aptamers

We used our recombinantly expressed Pap31 protein to identify candidate Pap31 protein-binding aptamers. We biotinylated our Pap31 protein using an EZ-Link Sulfo-NHS-Biotinylation Kit (ThermoFisher), then bound the biotinylated Pap31 to magnetic beads coated with streptavidin (Dynabeads M-270 Streptavidin)(ThermoFisher), following the manufacturer’s protocol. We used the bead-bound Pap31 to select Pap31 aptamer candidates via Sequential Evolution of Ligands by Exponential Enrichment (SELEX) using the X-Aptamer Selection Kit (AM Biotechnologies, Houston, TX, USA) following the manufacturer’s protocol for positive selection (using a library of oligonucleotides to bind the bead-bound Pap31), negative selection (using non-Pap31 functionalized beads), and in-solution selection (using a library of non-bead-bound oligonucleotides bound by non-bound biotinylated Pap31). We defined aptamer candidates as single-stranded DNA (ssDNA) sequences that demonstrated a binding affinity for the bead-bound Pap31 complex, as shown through the successful amplification of final pooled oligos. The amplification of oligonucleotide library aptamer candidates was performed via PCR using primers provided with the aptamer kit and a total reaction volume of 100 µL containing 1× PCR Buffer (10 mM Tris-HCl, pH 8.3, 50 mM KCl, 1.5 mM MgCl_2_), 2.5 mM of MgCl_2_, 0.2 of mM dNTPs, 0.4 µM of Forward Primer and 0.4 µM of Reverse Primer (X-aptamer selection kit, AM-Biotechnologies), and 1 unit of Taq Polymerase. The thermal cycler (C1000, Bio-Rad) conditions used were as follows: 94 °C for 1 min; 24 cycles at 94 °C for 30 s; 50 °C for 30 s; 72 °C for 1 min; a final extension at 72 °C for 3 min. The presence of amplified 50 bp SELEX library products was confirmed via gel electrophoresis using an agarose gel (2%) run in tris-acetate-EDTA (TAE) buffer (1×) consisting of Tris (40 mM), acetic acid (20 mM), and EDTA (1 mM) at 120 V for 30 min (Supporting Figure S3). The resultant enriched oligonucleotide pools were sequenced via Illumina high-throughput sequencing (performed by Raptamer Therapeutics, Houston, TX, USA). We identified six candidate Pap31 protein-binding aptamer sequences in this manner (sequences not shown due to Raptamer Therapeutics’ non-disclosure agreement).

### 2.3. Pap31 Binding of Fluorescein-Labeled Aptamer Candidates

We assessed the six candidate Pap31 protein-binding aptamers for their ability to bind to the Pap31 protein. The aptamers were synthesized with a 5′ fluorescein tag (IDT, Coralville, IA, USA) and reconstituted to 30 nM with nuclease-free water (Ambion, The RNA Company, Austin, TX, USA). We determined the Pap31-binding affinity of each candidate aptamer through electrophoretic mobility shift assays (EMSAs; use for aptamer validation shown previously [19,20]) using agarose gels (0.7%) with tris-borate-ethylenediaminetetraacetic acid (TBE) buffer (0.5×) consisting of Tris (65 mM), boric acid (22.5 mM), EDTA (1.25 mM), MgCl_2_ (5.0 mM), and Tween-20 (0.05%), with a pH of 8.0. Pap31 protein concentrations from 1 to 300 nM were tested with each aptamer. We imaged gels using a Gel Doc XR imaging system (Bio-Rad) and quantified Pap31 binding via lane analysis (ImageJ 1.53k; NIH, Bethesda, MD, USA) and Langmuir isotherm binding model fitting (Graphpad Prism 10.2.3; Boston, MA, USA). We selected the Pap31 aptamer candidate with the greatest Pap31 binding affinity to be used in our biosensor construction.

### 2.4. Pap31-Binding Aptamer Conformation Switching Design and Preparation

We modified the Pap31 protein-binding aptamer (identified as described in Section 2.2 and Section 2.3) to enable a conformation-switching approach in our E-AB biosensor design. Specifically, in this approach, which has been previously successfully used by our lab and others [12], the aptamer is modified to potentially exist in two iso-energetically favorable conformations, each of which produces a different, measurable electrochemical readout: (1) a target-binding conformation and (2) a non-target-binding (i.e., unbound) conformation. We determined predicted conformation folding patterns and associated energies using Quikfold Secondary Structure Prediction Routines [21]. To achieve two iso-energetically favorable conformations, aptamer sequence regions predicted to not participate in Pap31 binding interactions, as well as trailing 5′ and 3′ sequences, were systematically replaced (only using natural nucleobases and no poly-T tail), and then the resultant aptamer sequence was re-evaluated for secondary structure energetics. As part of this process, alterations were made to eliminate non-natural nucleotides in the parent aptamer. After confirming the energetics of the final sequence, we incorporated a disulfide linker molecule to the 5′ end of the oligonucleotide sequence (to enable binding of the aptamer to a gold-plated electrode surface) and appended an electrochemically active redox reporter group (methylene blue) to the 3′ end (to allow for a measurable change in electron exchange between the aptamer and the electrode surface). The resultant aptamer sequence used in the biosensor was 5′-(disulfide)-(thiol C6 SS)-CAC GAC GAA CCT GTG GCG CGC ACA GTC CAA GCC GGT GGG CCC ACA (methylene blue)-3′. This sequence was synthesized, purified via dual high-performance liquid chromatography (HPLC) (Biosearch Technologies, Petaluma, CA, USA), and reconstituted using nuclease-free water to 100 μM before being aliquoted into microcentrifuge tubes (5 μL each) and stored at −20 °C until use.

### 2.5. Electrode Preparation and Aptamer Appendment

The Pap31 protein-binding aptamer (prepared as described in Section 2.4) was appended to gold-plated electrodes, which were prepared as previously described [22]. To prepare the electrodes (CH Instruments, Austin, TX, USA; 2 mm diameter), potential organic surface contamination was first electrochemically removed from the electrodes via cyclic voltammetric scans in acidic and basic solutions. Specifically, the electrodes were initially placed in 0.5 M of sodium hydroxide and cycled from −0.4 V to −1.35 V, then placed in 0.5 M of sulfuric acid (H_2_SO_4_), held at 2.0 V for 5 s, held at 0.35 V for 10 s, and then cycled from 0.35 V to 1.5 V for 25 scans at a linear scan rate of 4 V/s. To etch away remaining contaminants, the electrodes were then placed in a solution of 0.01 M of potassium chloride and 0.1 M of H_2_SO_4_ and cycled from 0.2 V to 1.5 V for 40 scans. To confirm an absence of contaminants on the prepared electrodes, a final cyclic voltammetry scan in 0.05 M of H_2_SO_4_ was performed, cycling from −0.4 V to 0.1 V. To append the aptamer to the prepared electrodes, the aptamer was first activated by mixing 1:1 (2 µL of each component) with 1 M tris(2-carboxyethyl)phosphine hydrochloride (TCEP) in a microcentrifuge tube in the dark for 30 min, reducing aptamer disulfides to reactive thiols. The aptamer-TCEP mixture was then suspended in phosphate-buffered saline (PBS) with a pH of 7.4, consisting of 137 mM of NaCl, 10 mM of Na_2_HPO_4_, 1.8 mM of KH_2_PO_4_, 2.7 mM of KCl, and 1 mM of MgCl_2_, to a final volume of 100 µL, resulting in a 2 µM final concentration of activated DNA. The electrode was immersed in this solution for 1 h to append the aptamer. To prevent non-specific surface binding to the electrode, the surface was then backfilled overnight at 4 °C using 2 mM 6-mercaptohexanol, as previously described [22].

### 2.6. Pap31-Binding E-AB Biosensor Testing

Biosensor binding assays were performed using electrodes prepared as described in Section 2.5. An electrode’s surface was submerged in a PBS solution, as described in Section 2.5. Blank measurements were collected using PBS solution alone, with no target protein present, to generate a baseline for comparison after the addition of the target protein. Blank measurements were obtained by scanning the biosensor from −500 mV to 100 mV, with a 50 mV amplitude and a 0.5 mV increment over a range of frequencies (10 Hz to 250 Hz). Voltammetry data was gathered utilizing a WaveNano potentiostat (Pine Research Instrumentation, Durham, NC, USA) and AfterMath software (version 1.6.10523; Pine Research, Durham, NC, USA) in square wave voltammetry (SWV) mode. Next, a protein sample was added (all protein samples were prepared in PBS solution), the solution was allowed to equilibrate for ~7 min, and then measurements were taken across the same parameters as the blank measurements, with measurements taken over a range of protein concentrations (0 to 1.0 μM). Biosensor specificity was determined using the following off-target proteins: diluted bovine serum albumin (BSA) (0.5 μM) and recombinant ENOX2 protein (0.5 μM), the latter of which is a human serum-shed surface protein (purified by our lab as described previously [16]).

## 3. Results

### 3.1. Pap31-Binding Aptamer Screening and Selection

**Figure 2:**
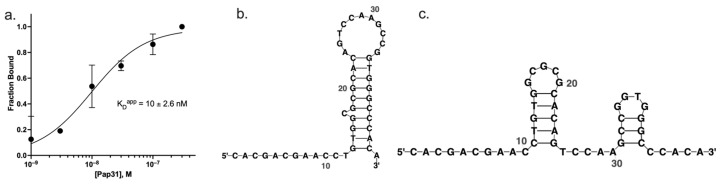
Pap31-specific aptamer binding and conformation switching. (**a**) Pap31 protein binding of the selected Pap31-specific aptamer via Pap31 titration in EMSAs, as described in Section 2.3. Aptamer binding affinity assessed via bound spot intensity and fit to a Langmuir isotherm model displayed robust and reproducible Pap31 affinity, with a K_D_^app^ of 10 ± 2.6 nM. (**b**,**c**) Secondary structure prediction and optimization of this aptamer were performed to create a structure with two isoenergetic states: (**b**) a disrupted binding interface (i.e., Pap31 non-binding state) and (**c**) an intact Pap31-binding motif (i.e., Pap31 binding state) (images generated with RNAcanvas software, https://rna2drawer.app/, accessed on 2 October 2024 [23]).

To identify a Pap31-binding aptamer for use in our E-AB biosensor, we performed SELEX using recombinant, purified Pap31 protein and an oligonucleotide library and tested the enriched oligonucleotides in Pap31 binding affinity assays (i.e., EMSAs). Pap31 protein purity was determined via SDS-PAGE (Supporting Figure S2), and only a purity ≥ 95% protein was used in downstream experiments. To identify DNA aptamers that efficiently bind the Pap31 protein, Pap31 was used in a SELEX process, as described in Section 2.2. Oligonucleotides pulled down from the oligonucleotide library using bead-bound Pap31 were confirmed through PCR amplification using oligonucleotide-flanking primers, followed by agarose gel electrophoresis and inspection for gel bands of the expected 50 bp size (Supporting Figure S3). The six most highly enriched Pap31-binding aptamer candidates were sequenced via high-throughput sequencing and then synthesized using fluorescein-labeled oligonucleotides to quantify their Pap31 binding affinity in EMSAs, as described in Section 2.3. Of these six aptamers, five showed minimal Pap31 binding affinity (Supporting Figure S4), and one displayed robust and reproducible binding affinity, with an apparent dissociation constant (K_D_^app^) of 10 ± 2.6 nM (Figure 2a; “Apt 1” in Supporting Figure S4). This aptamer, which showed the greatest affinity for Pap31 binding, was also the most highly enriched aptamer in the sequencing results of the SELEX process.

### 3.2. Pap31-Binding Aptamer Optimization to Enable Conformation Switching

The sequence of the aptamer identified in Section 2.3 was used as the basis for a Pap31-binding E-AB biosensor (Figure 1). The aptamer sequence was modified until two equally energetically favorable confirmations, (1) a Pap31 binding state and (2) a Pap31 non-binding state, were achieved, as described in Section 2.4 and shown in Figure 2b,c. Modifications to the candidate aptamer were made by interchanging nucleobases and predicting corresponding secondary structures using Quikfold [21] and an iterative method and process of aptamer modification previously described [24]. The final aptamer sequence selected for our biosensor design displayed isoenergetic binding and non-binding secondary structures when predicted using Quikfold.

### 3.3. Pap31-Binding E-AB Biosensor Sensitively and Specifically Detects Pap31

**Figure 3:**
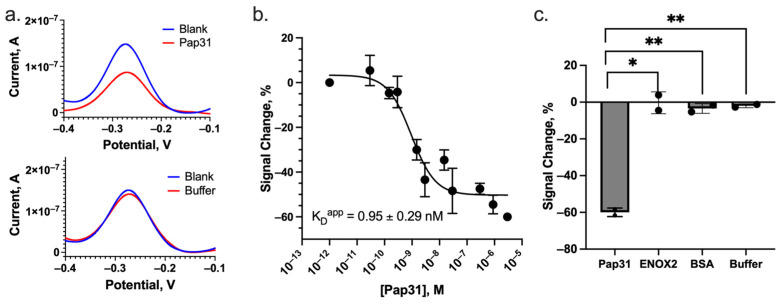
The Pap31-binding E-AB biosensor sensitively and specifically detected Pap31. (**a**) Raw current values of a prepared biosensor in buffer (“Blank”) challenged with the addition of 1 uM Pap31 (“Pap31”) (top panel) and alternatively challenged with the addition of buffer added in the same manner as used for Pap31 addition, but with no Pap31 protein included (“Buffer”) (bottom panel). (**b**) Titration of Pap31 protein against the biosensor equilibrated in buffer showed signal-off dose-responsive current changes, with a K_D_^app^ of 0.95 ± 0.29 nM. (**c**) This signal-off current change was not observed when the biosensor was challenged with non-target proteins ENOX2 (a secreted human protein biomarker; *p*-value < 0.03) or BSA (a major component of blood; *p*-value < 0.003) or serial addition of buffer vehicle only (*p*-value < 0.006). * *p* ≤ 0.05; ** *p* ≤ 0.01.

We quantified Pap31 binding sensitivity and specificity of our E-AB biosensor by titration of our biosensor with increasing concentrations of recombinant Pap31 protein, as described in Section 2.6. Our biosensor exhibited a characteristic signal-off response of approximately 60% decrease (Figure 3a, upper panel) in SWV current upon the addition of saturating Pap31 (final concentration of 1 μM, diluted in PBS buffer) to the biosensor, relative to the blank control (PBS buffer alone). To demonstrate that the observed signal change was specific to Pap31 addition and not due to an addition of buffer or change in buffer volume, we tested a buffer control (addition of buffer without Pap31 protein) and observed no significant signal change (Figure 3a, lower panel). Titrating varied concentrations of Pap31 allowed us to determine the apparent dissociation constant (K_D_^app^) of the E-AB biosensor for Pap31, which we found to be 0.95 ± 0.29 nM (Figure 3b; R^2^ = 0.89, Sy.x = 7.79), demonstrating extremely high sensitivity for Pap31. These measurements also demonstrated a very sensitive limit of detection (LOD) of 0.1 nM of Pap31. The LOD was calculated as 3.3 times the standard deviation divided by the slope of the calibration curve [25]. No significant current change was observed when the biosensors were challenged with the buffer alone (Figure 3c), suggesting that the observed current decrease is Pap31-specific. To further determine the specificity of the E-AB biosensor to Pap31 in the buffer, the biosensor was tested with off-target proteins: ENOX2 (0.5 μM), a human serum-shed surface protein also used as a clinical biomarker, and BSA (0.5 μM), a common blood carrier protein (Figure 3c). Challenging the Pap31 biosensor with ENOX2 and BSA did not similarly reduce the current readout compared to challenging with Pap31 protein (*p*-value < 0.03 and *p*-value < 0.003, respectively).

## 4. Discussion

Our E-AB biosensor presented here displays sensitive and highly specific detection in a buffer of Pap31, a biomarker in the outer membrane of *B. bacilliformis*, the causative pathogen for Carrion’s Disease. Our biosensor is extremely sensitive, with a K_D_^app^ of 0.95 ± 0.29 nM, exceeding the predicted clinically relevant concentration of Pap31 (~100 nM) [11,26]. Importantly, it displays this high affinity while displaying negligible signal change in buffer alone, BSA (a stand-in for common blood protein interferents), or a human serum-shed surface protein (ENOX2). While these are promising results, determining clinical relevance will require future work examining the performance of this E-AB biosensor in blood and other complex biological fluids. However, because other recent work has demonstrated the ability of this type of biosensor to function in environments as biologically complex as within living, ambulatory animals, there is good supportive evidence that future efforts focused on testing our biosensor in complex biological fluids ex vivo will prove fruitful [14]. All observations reported here were completed using a field-portable potentiostat to best reflect potential biosensor performance in applied settings. As there is a need for this class of biosensors to be robust, portable, and shelf-stable, efforts by other groups are underway to develop electrochemical instruments incorporating these biosensors that are able to meet all these requirements [9,27,28,29], including potentially smartphone compatibility [28], which will be an area for future development of our Pap31 E-AB biosensor. While our biosensor demonstrated minimal baseline changes in our experimental conditions, baseline drift of current in E-AB systems can be challenging. When our biosensor is further tested with complex biological fluids in the future, we may pursue potential solutions previously shown to correct for baseline drift, such as kinetic differential measurement or physical surface protection [13,30]. Overall, the highly sensitive Pap31-binding E-AB biosensor presented here may aid in the future development of diagnostic tools for improved rapid and sensitive diagnosis of Carrion’s Disease through detection of the causative bacteria in vivo or in water supplies.

## Data Availability

The original contributions presented in the study are included in the article/Supplementary Materials; further inquiries can be directed to the corresponding author.

## Supplementary Materials

The following supporting information can be downloaded at: https://www.mdpi.com/article/10.3390/s24227295/s1, Figure S1: Plasmid map of the Pap31 protein recombinant expression vector; Figure S2: Pap31 protein purity assessed via 10% SDS-PAGE following purification via FPLC; Figure S3: Agarose gel electrophoresis (2%) of enriched aptamer candidates (expected size 50 bp), following X-Aptamer selection protocol; Figure S4: Electrophoretic mobility shift assay binding data for putative Pap31 aptamer candidates.
