# Supplementary material for: Electrochemical Aptamer-Based Biosensor for Detecting Pap31, a Biomarker for Carrion’s Disease
Source: Sensors (Basel). 2024 Nov 15;24(22):7295. doi: 10.3390/s24227295 (PMC11598354; doi:10.3390/s24227295)
Supplement: Supplementary file 1 [file sensors-24-07295-s001.zip › sensors-3292198-supplementary.pdf]

Supporting Information for

Electrochemical Aptamer-based Biosensor for Detection of Pap31, a Biomarker for Carrion's Disease

Keaton Silver [1], Andrew Smith [2], Haley V. Colling [1], Nico Tenorio [1], Teisha J. Rowland [3], and Andrew J. Bonham [1,*]

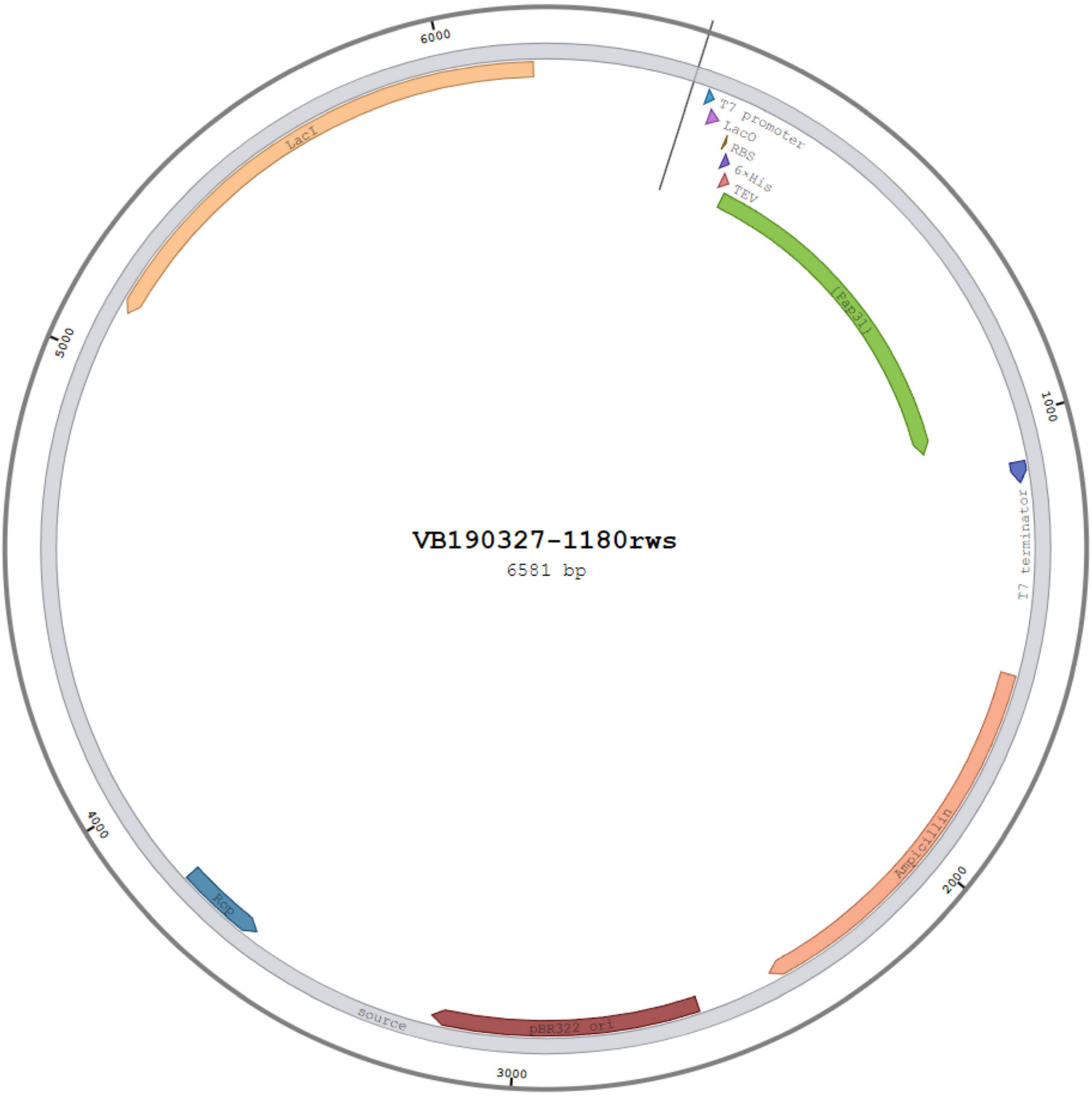

Supporting Figure S1. Plasmid map of the Pap31 protein recombinant expression vector (VectorBuilder, Chicago, IL). Plasmid features 6xHis affinity tag at the N-terminus of the construct.

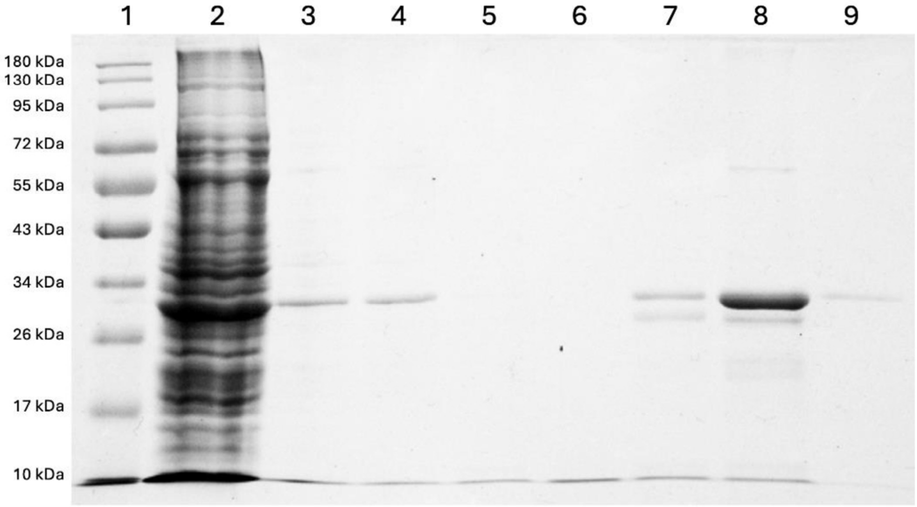

Supporting Figure S2. Pap31 protein purity assessed via 10% SDS-PAGE following purification via FPLC. Lane 1: Protein marker ladder (New England Biolabs #P7719; Ipswich, MA, USA); Lane 2: Lysate of *E.coli* induction; Lanes 3 – 6: FPLC wash steps using wash buffer pH 6.3; Lanes 7-9: FPLC elution steps using wash buffer pH 5.9. Protein from lane 8 was pooled and quantified via band density at >95% purity for use in subsequent experiments (Image J 1.53k; NIH, Bethesda, MD, USA).

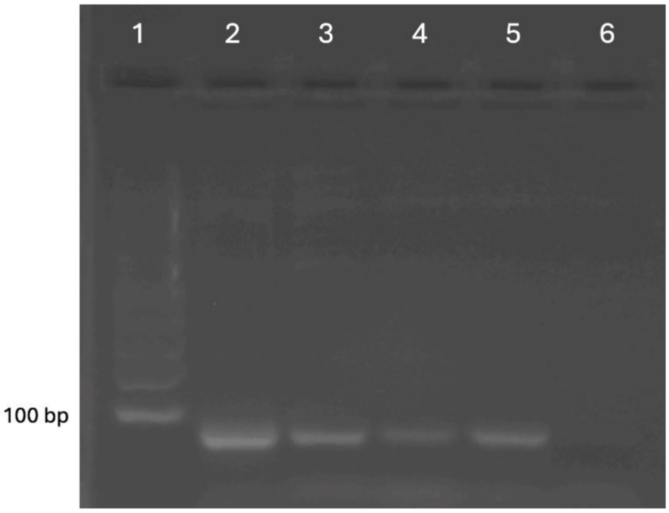

Supporting Figure S3. Agarose gel electrophoresis (2%) of enriched aptamer candidates (expected size 50 bp), following X-Aptamer selection protocol. Lane 1: 100 bp molecular ladder (New England Biolabs #N3231; Ipswich, MA, USA); Lanes 2-6: PCR product of aptamers selected against different targets; Lane 2: Positive selection control; Lane 3: Pap31 selected aptamers; Lane 4: Pertactin selected aptamers; Lane 5: Glycopeptidolipid selected aptamers; Lane 6: Negative selection control.

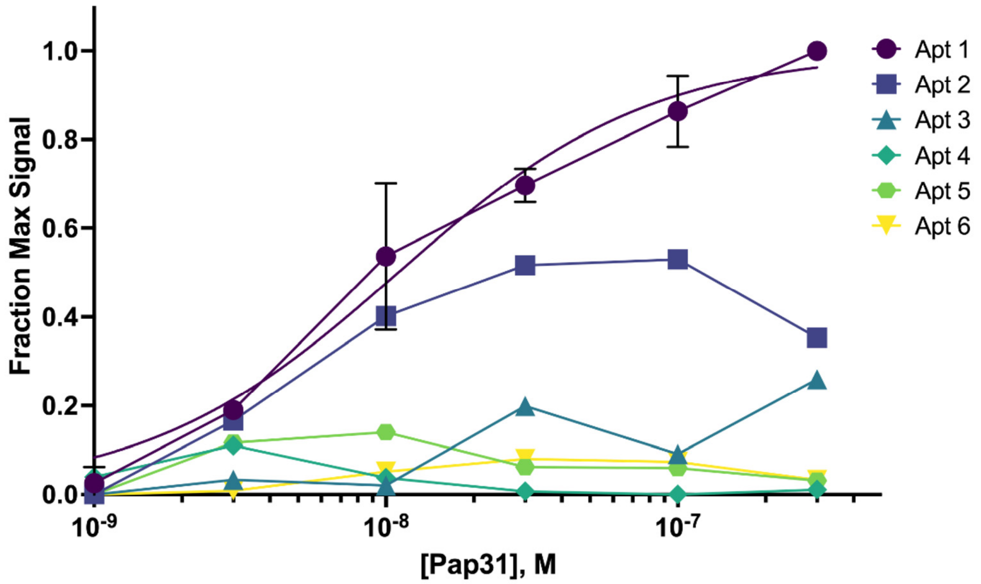

Supporting Figure S4. Electrophoretic mobility shift assay binding data for putative Pap31 aptamer candidates. Fluorescein-labeled aptamer candidates were assessed on agarose gels (0.7%) with tris-borate-ethylenediaminetetraacetic acid (TBE) (0.5×) consisting of Tris (65 mM), boric acid (22.5 mM), EDTA (1.25 mM), $MgCl_2$ (5.0 mM), and Tween-20 (0.05%), pH 8.0. Pap31 protein concentrations from 1 to 300 nM were tested with each aptamer. The signal was normalized to maximum integrated spot intensity across all aptamers tested. Only aptamer candidate 1 displayed reliable binding, with a K_D^{app} of 10 ± 2.6 nM.
